# Co-design of a theory-based implementation plan for a holistic eHealth assessment and decision support framework for people with dementia in care homes

**DOI:** 10.1177/20552076231211118

**Published:** 2023-11-28

**Authors:** Juliet Gillam, Catherine Evans, Jesutofunmi Aworinde, Clare Ellis-Smith, Jamie Ross, Nathan Davies

**Affiliations:** 1 Florence Nightingale Faculty of Nursing, Midwifery & Palliative Care, Cicely Saunders Institute of Palliative Care, 4616 King's College London, London, UK; 2 Brighton General Hospital, Sussex Community NHS Foundation Trust, Brighton, UK; 3 Centre for Primary Care, Wolfson Institute of Population Health, Barts and The London School of Medicine and Dentistry, 4617 Queen Mary University of London, London, UK; 4 Centre for Ageing Population Studies, Research Department of Primary Care and Population Health, 4919 University College London, London, UK

**Keywords:** Dementia, eHealth, implementation, care homes

## Abstract

**Background:**

Despite positive findings around the use of eHealth in dementia care, it is rarely translated into routine practice. This can be facilitated by early involvement of end-users in the development of an implementation plan. This study aimed to co-design strategies to implement an eHealth intervention, the EMBED-Care Framework, to support assessment and decision-making for people with dementia in care homes.

**Methods:**

A qualitative co-design method was applied through a series of workshops. Participants included family carers and health and social care practitioners. People with dementia were included through a series of stakeholder engagement meetings. The workshops focused on co-developing strategies in response to identified determinants of implementation. A codebook thematic analytic approach was taken, guided by the Normalisation Process Theory (NPT).

**Results:**

Three workshops were conducted from July 2021 to November 2021, attended by 39 participants. Three overarching phases of implementation were identified which aligned with the constructs of the NPT: (a) incentivising adoption of the Framework, which requires promotion of its benefits and alignment with recommendations for good quality dementia care to engage stakeholders, relating to ‘coherence’ and ‘cognitive participation’ constructs; (b) enabling its operation, which requires ensuring compatibility with care home processes, provision of training and support from ‘champions’, relating to ‘collective action’; (c) sustaining use of the Framework, which requires monitoring of implementation and appraisal of its effects, relating to ‘reflexive monitoring’.

**Conclusions:**

We have developed a multi-strategy, theoretically driven plan to implement eHealth to support assessment and decision-making for people with dementia in care homes. Successful implementation requires incentivisation to adopt, ability to operate and motivation to sustain use of eHealth. The plan is strengthened through collaborating with end-users to increase its value, credibility and real-world relevance. The theoretically informed strategies target mechanisms of the NPT, demonstrated to shape the implementation process and outcomes, ready for testing.

## Introduction

Around 55 million people are currently living with dementia and this is estimated to increase to approximately 153 million by 2050.^1^ To meet the rising care needs for this growing ageing population, there is increasing demand for long-term care. Internationally, the term ‘care home’ refers to long-term care provision for adults who require 24-h assistance with personal care and activities of daily living.^2^

In the UK, care homes are the largest provider of end-of-life dementia care.^3^ Seventy percent of those however are residential,^4^ meaning they have no onsite physician or nursing staff. Rather, external health care services manage residents’ often complex multiple health needs associated with dementia and multimorbidity.

Access to these external services is inequitable, leaving thousands of people with dementia in the UK lacking the support and care they require.^5^ This is driven by the artificial divide between health and social care and an unequal distribution of healthcare resource nationally. Integrating health and social care is widely acknowledged to improve person-centred treatment outcomes for older people with complex needs.^6^ Whilst integration is a governmental priority in the UK and some progress is evident,^7^ these two entities remain separate.

eHealth, defined as ‘health services and information delivered or enhanced through the internet and related technologies’,^8^ can facilitate this integration and provide communication routes with external healthcare professionals that care homes require. During the COVID-19 pandemic, use of eHealth grew exponentially to allow for remote access to healthcare in the community, with rising demands and restrictions on visiting for infection control and prevention.^9^ eHealth enabled access to external specialist input for residents in care homes, remote monitoring and the crucial continuation of information-sharing between services to inform treatment and care planning.^10^

Positive findings regarding effectiveness of eHealth, however, do not guarantee its successful uptake. It is estimated that up to 80% of electronic health record initiatives fail to be implemented.^11^ Barriers to successful adoption of eHealth in the care home are common and wide-ranging, and include incompatibility between the intervention and care home processes, an overly complex intervention protocol and inadequate training and support for users.^12^

A key strategy in mitigating some of these barriers, and to promote uptake of eHealth is to co-design the intervention and implementation plan with users and relevant stakeholders.^13^ Co-design necessitates a shift in the traditional power balance between researchers and end-users to ensure collective ownership, equal participation and legitimate shared decision-making.^14^

Despite being shown to facilitate implementation,^15^ involving stakeholders prospectively in the development of an implementation plan is rare.^16^ More frequently, stakeholders are consulted retrospectively to review the implementation plan, which can impede research impact.^17^

### The intervention: the EMBED-Care Framework

In order to meet care demands, a multi-disciplinary model of care is required. The Empowering Better End-of-life Dementia-Care (EMBED-Care) Framework aims to optimise the provision of palliative dementia care. The Framework comprises the Integrated Palliative care Outcome Scale for Dementia (IPOS-Dem), a comprehensive holistic assessment tool to facilitate identification of symptoms and concerns,^18^ linked with evidence-based decision support tools for managing symptoms common in dementia,^19^ with resources to support use. If a concern is identified on the IPOS-Dem, the user will be directed to a decision support tool, to support management of the identified need and care delivery. The Framework is delivered via an app or website.

### Aim

To identify user requirements for a holistic eHealth assessment and decision support tool (the EMBED-Care Framework) with people affected by dementia in care homes and their health and social care practitioners, and co-design its implementation plan.

## Methods

### Study design

A co-design study which drew upon qualitative methods was conducted through a series of workshops and stakeholder engagement meetings. This study formed the second component of a wider sequential study to develop and evaluate the feasibility of the implementation plan for the EMBED-Care Framework. It was informed by findings from a systematic review of the published evidence on the determinants of implementation for eHealth interventions, for holistic assessment and decision-making for people with dementia in care homes.^12^ We used the consolidated criteria for reporting qualitative studies (COREQ)-32 item checklist to facilitate transparency and promote comprehensive reporting^20^ (see Supplemental Table 1).

### Co-design method used to develop implementation plan

The co-design workshops drew on O’Cathain's ‘Partnership Approach’ to intervention development.^21^ Partnership synthesises the key elements of co-design, co-creation and co-production. These are distinct but overlapping approaches to healthcare improvement that share the fundamental thread of collaborating with stakeholders in the design and production of research.^14, 22, 23^ O’Cathain proposes six steps, which were used to guide the co-design process (see Table 1).

**Table 1.** Application of the six steps of O’Cathain's ‘Partnership Approach’ to this study.

O’Cathain's six steps	Application in this study
1. Identify a team of end-users and relevant stakeholders	Health and social care staff, family carers, people with and affected by dementia and PPI members were identified
2. Share knowledge and experience and understand the current problem	The problem was detailed in the 1st workshop, informed by the determinants of implementation identified in the systematic review^12^ and discussion to understand experiences and wider knowledge from the ‘real-world’
3. Co-create by listening to all voices	All participants were encouraged to contribute to the discussions, facilitated by use of smaller virtual break-out rooms and a series of engaging tasks, including use of electronic interactive whiteboards
4. Co-design the solution using qualitative research and prototypes	Workshops were guided by a topic guide created from the systematic review^12^ and the Normalisation Process Theory.^45^ Through discussion, we refined areas of importance, achieved consensus on implementation requirements and identified suitable implementation strategies to overcome the barriers identified
5. Build the solution in small action groups using relevant expertise	All participants in the co-design workshops had direct experience of caring for at least one person with dementia, which was integral to development of the plan. The workshops were complemented by engagement activities involving consultations with people affected by dementia about the proposed implementation plan
6. Measure outcomes together	This will occur in the feasibility study

### Underpinning theory

**Figure 1.**
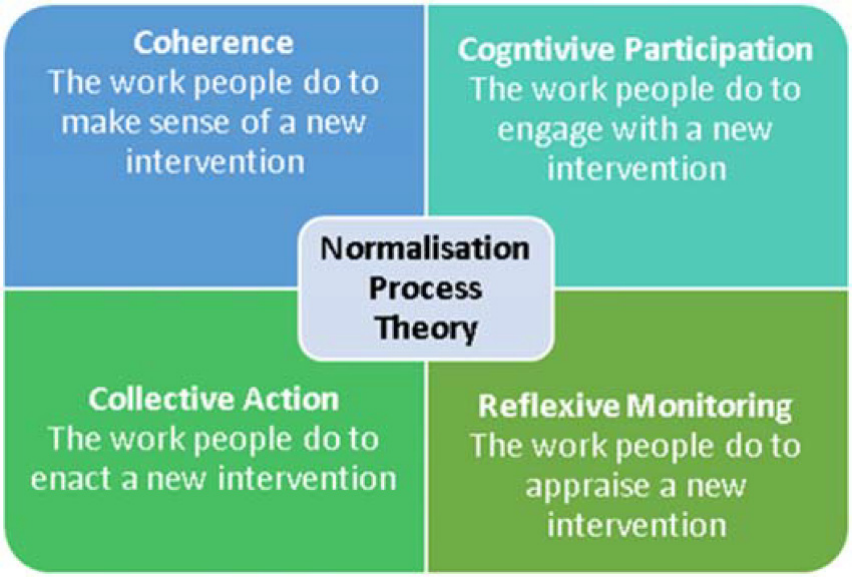
Normalisation Process Theory constructs.^24^

The study was underpinned by the Normalisation Process Theory (NPT); an implementation theory concerned with explaining the collective and individual work which people do to embed interventions in routine practice.^24^ It identifies four key mechanisms which have been empirically demonstrated to shape together the social processes of implementation, and promote the adoption of this kind of eHealth intervention^24^ (see Figure 1).

### Participants

Research participants comprised family carers, current and bereaved carers (including close friends) and health and social care practitioners with a range of roles to reflect the multi-disciplinary team caring for people affected by dementia. A planned sample of up to 40 participants across the workshops was estimated to generate sufficient information power, by considering the breadth of the study aims, participant specificity, theoretical support and plans for analysis.^25^ Participants were recruited purposively, monitoring for age, gender, ethnicity and role to maximise sample diversity, and enable a wider understanding of different perspectives and lived experiences.

Various sources were used to recruit participants from groups across the UK, to allow for a more representative sample and to consider differences that may arise in rural versus urban areas. These included charity and voluntary sector groups and carer networks; health and social care practitioners; and care home staff identified via The National Institute for Health Research Enabling Research in Care Homes (NIHR ENRICH) Network regional leads. Some participants had been involved in previous projects, and were known to the researchers. As the workshops were conducted during COVID-19, participants were initially approached via email, and written informed consent was obtained from all participants.

Eligibility criteria: People living with dementia who:

- Have a clinical diagnosis of any type of dementia (as confirmed by participant themselves or their family carer)
- Have mental capacity to provide informed consent
- Are able to read and speak English

Carers who:

- Are a current or bereaved carer of someone with dementia
- Can provide informed consent
- Are able to read and speak English

Professionals and practitioners who:

- Have a caring role either health or social care, for people with dementia
- Experience of developing tech in healthcare
- Are able to provide informed consent
- Are able to read and speak English

### Co-design workshops: content and structure

**Figure 2.**
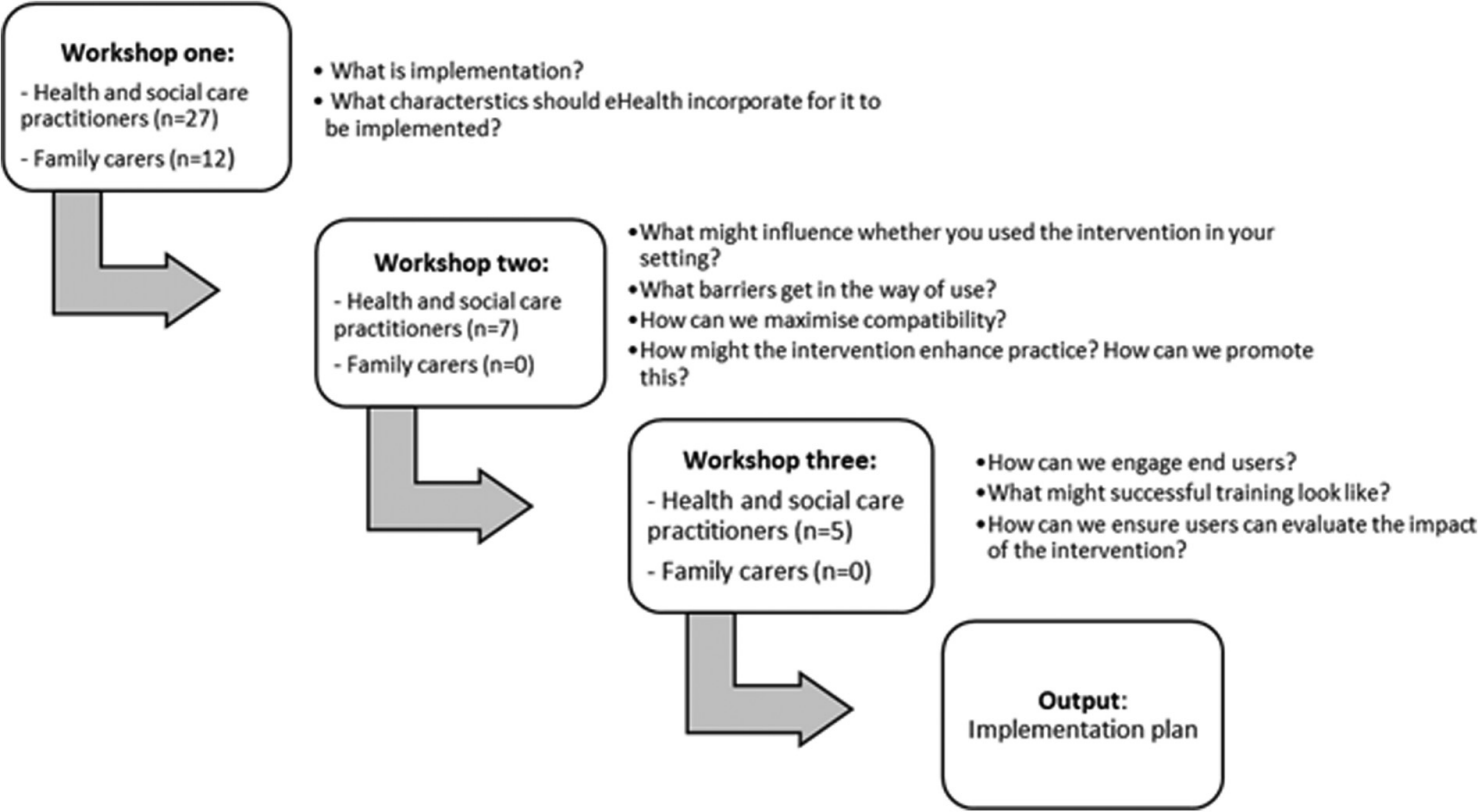
An overview of the key topics covered in each workshop.

Three co-design workshops were held over a period of six months, led by different members of the research team (JG, JA, ND; Figure 2). Each workshop was held via the video-conferencing software ‘Zoom’ and lasted up to two hours. At the start, each researcher introduced themselves and their reasons for conducting the research. The first workshop was attended by healthcare professionals and family carers of people with dementia in both the care home and community settings. The subsequent two were specific to the context of a care home, and were attended solely by practitioners who had direct experience of caring for somebody with dementia in a care home.

Questions asked in the workshop were informed by the key determinants of implementation identified in the systematic review.^12^ Although it provided a set of factors relevant to implementing eHealth in care homes, these were derived from many papers from different countries, utilising a range of eHealth interventions. It was therefore necessary to pursue these potential determinants in relation to the EMBED-Care Framework and identify which of them were relevant for implementing this specific type of eHealth in this context. Uncertainties generated in the review were also pursued, for example, around how to best provide training, build notifications into the app and involve family members in care decisions.

Additional questions to guide discussion were developed based on the four constructs of the NPT. This was to encourage the identification of theory-based determinants of implementation, and the generation of corresponding implementation strategies (see Supplemental Table 2 for an overview of the questions asked in each workshop).

#### Workshop one: identification of core features for implementation and intervention components

The aim of the first workshop was to explore participants’ initial reflections on an early prototype of the Framework. Small group discussions were used to revise the Framework prototype. Participants considered what potential components were priorities to facilitate its use in the care home setting. The group was presented with features pertaining specifically to eHealth which had been found to influence implementation success from the systematic review.^12^ These included customizable features, inbuilt reminders for use and compatibility with technology currently in use.

#### Workshop two: integration with current practice

Workshops two and three focused specifically on co-designing the implementation plan for the Framework, to be delivered via an app. Discussion centred around the constructs of the NPT, and how these could be promoted and operationalised in practice. The second workshop focused on exploring how we could ensure fit between the Framework and the care home setting to promote ‘collective action’. This was chosen as the starting point as the first iteration of the NPT was focused exclusively on this construct, and the practical determinants of eHealth implementation were amongst the most salient and influential in the systematic review. Discussion focussed on identifying strategies which could ensure compatibility between the EMBED-Care Framework and current processes around assessment and decision-making in the care home setting, and overcome potential barriers to use. The group also considered how to promote ‘coherence’ around the Framework, and how its anticipated benefits could be promoted in the real world.

#### Workshop three: implementation requirements and resources

The final workshop comprised two parts. The first part centred on maximising engagement of care home staff, people with dementia and family carers with the Framework to enhance ‘cognitive participation’. In breakout rooms, strategies explored included use of ‘champions’ and tailoring implementation protocols to the context of care homes. As training was the most salient factor identified in the systematic review, we also asked specific questions around what might be required to support use of the Framework, and for training to be delivered effectively.

In the second part of the workshop, the group discussed approaches to ‘reflexive monitoring’; evaluation and appraisal of the Framework to ensure that users are aware of its impact. The groups were asked to consider what feedback might be helpful for individuals to receive, to motivate and sustain use of the Framework and how this might be provided to best facilitate implementation.

### Patient and public stakeholder engagement meetings

We conducted a series of stakeholder engagement meetings with people affected by dementia. This enabled consultation with people living with mild dementia, including young onset dementia, and family carers. Workshops were held with established groups including a Pathways Group from the Dementia Engagement and Empowerment Project (DEEP) and the EMBED-Care Personal and Public Involvement (PPI) study reference group.

Prior to the co-design workshops, the groups helped advise on our planned content for the co-design workshop. Subsequently, they helped us to make sense of data generated in the workshop discussions and inform iterations of the implementation strategies before presenting them back to the co-design groups.

### Data analysis

Co-design workshops were audio recorded and detailed notes were taken during each of the sessions by a scribe. In between workshops, a rapid thematic analysis^26^ was conducted to identify any determinants of implementation or uncertainties which would require further thinking and development in subsequent workshops.

After the final workshop, a codebook thematic analysis was undertaken, following guidance from Braun and Clarke.^27^ After a period of data familiarisation, a codebook was deductively developed in NVivo,^28^ guided initially by the constructs of the NPT. This was then inductively revised to account for overlap between the theories’ four constructs, and the phases of requirements which were identified. Subsequently, the codes were sorted by merging related codes into potential themes. These were then iteratively reviewed, reorganised and defined to form the final set of themes through team discussions.

Initial coding was conducted by one researcher (JG), with sense-checking and discussions at each subsequent stage of refinement by two others (CE and ND). As the data collected was qualitative, it was vital to apply a rigorous approach to allow for confidence in the findings and enhance reliability and trustworthiness.^29^ This was partially achieved through application of the NPT^24^; employment of relevant theory is an established quality indicator of rigour in qualitative research.^30^ Further, reflexive practice plays an instrumental role in achieving rigour and quality.^31^ Throughout the process, the primary researcher (JG) reflected on her own social and cultural experiences as a white female PhD fellow with a background in Psychology and mental health, and kept field notes to monitor and interrogate initial assumptions, to optimise data analysis.

## Results

### Participant characteristics

A total of 39 participants encompassing a mix of health and social care practitioners (n = 27) and family carers (n = 12) were involved in co-designing the implementation plan across the three workshops (see Table 2 for details). No participants withdrew their data.

**Table 2.** Participant characteristics.

Characteristic	Practitioners (n = 27)	Family carers (n = 12)
Age		
Mean (SD)	46.1 (10.2)	62.6 (9.2)
Sex		
Male (n)	4	1
Female (n)	23	11
Ethnicity		
White, British or Irish White Other	19	12
Asian (Pakistani, Sri Lankan)	3	0
Black (African)	2	0
Role		
Primary Care (including GPs and nursing staff)	4	
Specialist Care (including palliative nurse specialists, consultant psychiatrists and palliative consultants)	13	
Social care (including care home managers and support workers)	4	
Others (including improvement, education, experience and PPI leads)	6	
Relationship to person with dementia		
Spouse		3
Child		6
Niece		2
Granddaughter		1

### Findings: requirements to implement the EMBED-Care Framework

Three overarching requirements for uptake of the EMBED-Care Framework were identified across the three workshops. These relate to three phases of implementation and are underpinned by the NPT constructs: (a) incentivising adoption of the Framework relating to ‘coherence’ and ‘cognitive participation’; (b) enabling operation of the Framework relating to ‘collective action’ and (c) sustaining use of the Framework relating to ‘reflexive monitoring’. The full illustrative quotes are presented in full in Supplemental Table 3.

#### Phase one: Incentivising adoption of the Framework

To attain coherence around the Framework and sufficient cognitive participation from stakeholders, discussion focused on its purpose and how it might prove advantageous to current practice to provide impetus for engagement.

##### Incentivizing residents and their family

Potential benefits to residents centred on how using the Framework could optimise the quality of care they receive. A key pillar of the Framework is to enhance person-centred care, with emphasis on the needs of the individual. Participants recognised this strength, detailing, for example, the Framework ‘allows the person living with dementia to be at the centre’ (GP, WS3). It also has the potential to improve identification of residents’ concerns (Hospice education lead, WS2); particularly regarding their ‘psychological welfare’ which participants felt, although vital, is often overlooked in care homes for residents towards the end of life (Hospice education lead, WS2).

Participants acknowledged the importance of involving families in delivering good quality person-centred care. Providing them with access to information and updates about their loved one is a requirement for use of the Framework, and essential for their cognitive participation (Family carer, WS1). Debate centred around the extent to which families should receive notifications; some participants thought it was key to alert the family with every change in presentation, whilst others felt this would be too frequent and potentially overwhelming, and a barrier to sustained use. A tailored ‘horses for courses’ approach to the needs of each individual family was proposed as a possible solution (Nurse specialist, WS1).

The Framework was also considered to have the potential to facilitate integrated working and communication about the resident between the care home and external health services. This feature promoted coherence and cognitive participation amongst the workshop participants, particularly in light of their experience of COVID-19 (Care home manager, WS2). Care staff expressed concern about the challenge posed to care homes’ access to healthcare in ‘lockdowns’. The impact this ‘backlog’ had on the quality of care and treatment residents received was felt to be of particular importance, when routine in-person input from general practitioners (GPs) and community nursing teams was disrupted (Consultant psychiatrist, WS2).

##### Incentivising health and social care practitioners

Promoting the Framework's potential benefits to health and social care practitioners was identified as vital. Successful uptake required a ‘whole-team approach’ from a multi-disciplinary network of stakeholders. Participants had opposing viewpoints as to whether the focus should be on ‘convincing the manager’ (Palliative care nurse specialist, WS3) or whether to target those ‘on the floor’ who would be delivering the care, for optimal cognitive participation (Advanced clinical practitioner, WS2).

The impact that participating in the EMBED-Care study could have on care home staff's personal development and workload was discussed as an incentive for participation. Empowering staff to raise a concern to a healthcare professional through providing the shared language required to ‘articulate their concerns’ was seen as a key benefit (Advanced clinical practitioner, WS3). Due to COVID-19 lockdown restrictions, the role of care home staff became increasingly comprehensive, with additional demands depleting emotional and cognitive reserves. Ensuring benefits are known to staff is key to avoid ‘change fatigue’—staff resistance to adopt change because of failure to observe a positive difference (Consultant psychiatrist, WS2). This was acknowledged by participants to be a key potential barrier to staff engagement.

Participants suggested that engagement with the Framework will be largely influenced by how aligned it is with recommendations from healthcare standards and external governing bodies for delivery of good quality dementia care. Emphasising to potential users where embedding the Framework in their practice supports these recommendations and can address national priorities, for example, around encouraged use of digital assessments, can ‘appeal to their ambitions’ and act as a lever for adoption (GP, WS3). However, changes at a national level can also act to inhibit uptake of the Framework, even if the change supports use of the Framework. In England, there are currently planned changes to healthcare commissioning structures, to support a more integrated way of working between services. Despite this being synchronous with the principles of the Framework, participants reported apprehension around adopting a new model of care, whilst operating in an already changing and uncertain landscape (Hospice education lead, WS2).

##### Barriers to adoption

Despite many advantages, digital delivery of healthcare poses a unique set of challenges. A fundamental issue with implementing an app with this population relates to ‘triggering anxieties’, and users’ unfamiliarity, or inability to utilise technology (Nurse specialist, WS1). This undermines coherence around the Framework and the incentive to engage. Participants also acknowledged the care becoming ‘very depersonalized’ when technology is involved (Nurse, WS1), and the negative impact it has on concentration. Further concerns pertained to residents’ ability to consent to data sharing, information governance and meeting General Data Protection Regulation (GDPR) requirements (GP, WS1).

#### Phase two: Enabling operation of the Framework

The second theme relates to what is required for collective action, and to practically enact a new way of working following initial incentivisation. This focuses primarily on the Framework's compatibility with how each care home operates, and functional strategies which could facilitate its use.

##### Compatibility between the Framework and care home

Participants stressed the importance of the EMBED-Care Framework ‘avoiding duplication’ and being easily interoperable with current practice for successful implementation (Hospice education lead, WS2). It cannot disrupt duplicate workflow; rather, it must enhance current assessment and decision-making procedures, and optimise the way care is delivered. A fundamental obstacle to achieving this is the huge disparity that currently exists between care homes with regards to current routine care procedures, and their variable access to digital infrastructure such as Wi-Fi and tablets to support care delivery (Nurse specialist, WS1). Communication methods with individuals outside the home including families and affiliated health services such as GPs—who themselves use a variety of different electronic health systems—are also ‘very sketchy around the country’ (Hospice education lead, WS2).

Attempting to accommodate the variations between care provision at both a local and national level poses a real challenge to implementation of the Framework. One of the most salient findings from the systematic review^12^ was around the importance of ensuring eHealth is adaptable, in order to optimise compatibility. How to tailor the Framework to the local needs of each care home and resident, was therefore pursued in the workshops (Nurse specialist, WS1). This pertained to how regularly the assessment should be completed, by whom, on what platform and how the information is subsequently shared. Implementing the Framework in care homes within the same community following the same protocols and sharing the same external staffing structures, was also suggested as a means to overcome issues of disparity (GP, WS3).

##### Facilitating the transition to digital health

Participants recognised the challenge that changing to eHealth from a primarily paper-based system would pose. The review elucidated several practical strategies demonstrated to ease this transition, which were presented to the group. As discussed, there is much variation in provision of resource within care homes, so initially it must be ascertained whether individuals in each have access to the necessary digital infrastructure to utilise the Framework, and ensure it is provided. To initiate use, participants advocated for inclusion of alerts built into the app to notify users if a concern has been identified or a change has been observed, and subsequently to escalate these concerns (Family carer, WS1). The importance of developing a digital interface which is consistent over time, ‘very simple’ and easy to use for both carers and people with dementia (Family carer, WS1), was deemed particularly important if it is going to be adopted, with overly complex technology and software updates being acknowledged as a barrier to use. Offering comprehensive technological support was recognised to be essential, along with additional resources and features built into the app to assist with use—such as a glossary of keywords (Family carer, WS1).

##### Champions

Workshop participants unanimously advocated for the use of ‘champions’ to facilitate implementation. The role of the champion is to drive implementation of the Framework in the care home, and to support staff members to prioritise and perform new or additional roles to deliver the change in care practice. Delivering training to other staff and ‘constant monitoring’ of change are also key elements of quality improvement and form part of a champion's responsibilities (Hospice clinical lead, WS2). It was felt that using a champion model was of particular importance in the care home setting, where ‘significant turnover of staffing’ is common (GP, WS2).

##### Training

To maximise uptake and ensure collective action, sufficient training must be provided for all users. Discussion centred around what constituted good training, and what has previously worked well. The consensus was that offering a ‘blended-approach’ (Hospice education lead, WS3), through a variety of training methods including instructional videos and in-person training, was the best format for busy care home staff. Training should be simple and accessible in ‘bite-size chunks’ (Advanced clinical practitioner, WS3) and offered to all staff members with the incentive of ‘career progression’ (GP, WS2).

The potential value of training to be used as a vehicle to foster integration between health and social care professionals was also discussed, to promote ‘understanding of each other's challenges’ (Hospice education lead, WS2) and an appreciation of each other's roles. This can be achieved by delivering it to a mixed, multi-disciplinary group including care staff of different grades and expertise, helping overcome traditional hierarchies between health and social care professionals (GP, WS2).

#### Phase three: Sustaining use of the Framework

The third and final theme relates to what will be required for individuals to sustain use of the EMBED-Care Framework. Reflexive monitoring—being able to observe and appraise the outcomes that implementing a new way of working is having—is key for continued engagement.

##### Providing feedback to stakeholders

Participants suggested various forms of feedback which would be helpful for individuals to receive—both as a desirable outcome of using the framework, and to ‘feel the benefit’ (Nurse specialist, WS1) using the Framework can have. This centred around data which shows a positive change or impact resulting from its use, either on clinical outcomes for residents, safety of care delivery or workload for practitioners (Clinical nurse specialist, WS3).

Several ways in which feedback could be made accessible were suggested. This included developing graphs illustrating both the change in symptoms over time as a result of using the Framework (Clinical nurse specialist, WS3) demonstrating enhanced care delivery, and staff adherence to it (GP, WS3). Using online platforms such as Facebook and care home forums as vehicles through which to promote the positive impact of the Framework, and circulating this sort of data, was recommended by participants (Hospice education lead, WS3).

##### Continuous monitoring of implementation

Groups discussed that sustaining a change in behaviour requires ongoing active monitoring of the implementation process and strategies. This can be achieved by taking ‘small steps’ initially by rolling the Framework out on a small scale, to allow for any necessary amendments to be made to the implementation plan ahead of widescale roll-out (Care home activities coordinator, WS2). Making adherence data available was also proposed as a way of ‘monitoring and changing habits’ (Hospice education lead, WS2).

## Discussion

### Principal findings

**Figure 3.**
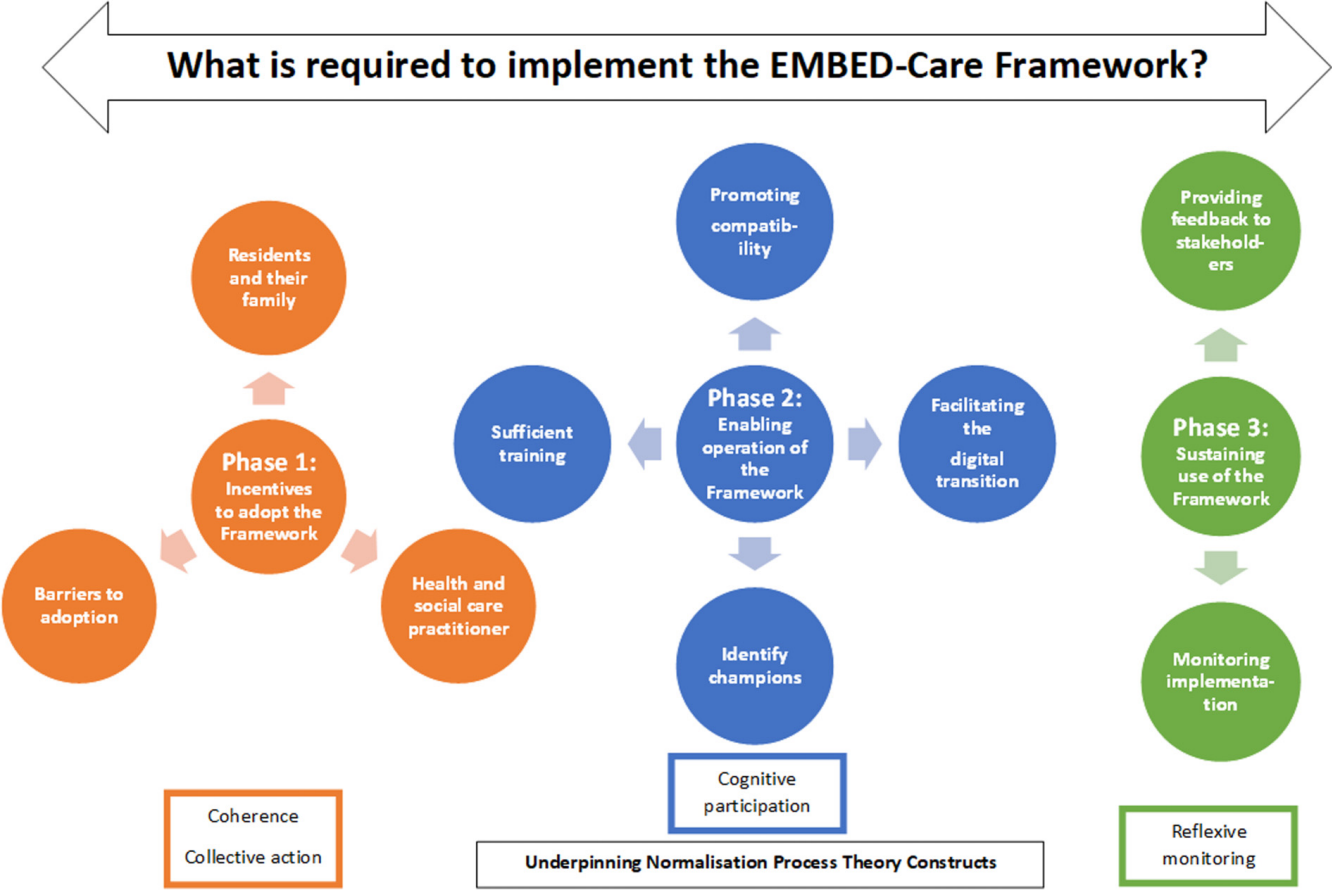
Phases of implementation of the EMBED-care Framework underpinned by NPT constructs.

Underpinned by the key concepts of the NPT, we have inductively revised the theory's four constructs to generate three overarching themes, which encapsulate the key mechanisms of change required to implement the EMBED-Care Framework at three distinct phases of implementation. For successful implementation, individuals must be incentivised to adopt the Framework, able to operate it and motivated to sustain its use. The phases are associated with the four different constructs of the NPT (see Figure 3). Each has its own implementation requirements and corresponding strategies to meet these requirements. Revision of the constructs was to reflect the overlap and temporal nature of the NPT constructs when applied in this context and to account for the phases of implementation identified in the data, thereby increasing applicability of the theory to this population, setting and intervention type.

The impact of COVID-19 was unsurprisingly a common feature of discussion. The effect of the pandemic on care homes was monumental: it is estimated that in the UK, deaths of care home residents accounted for 40% of all COVID-19-related deaths.^32^ The concern was the ongoing impact, its effect on care homes’ access to healthcare and staff workload would lessen individuals’ incentive and capacity to engage with new ways of working, such as the EMBED-Care Framework.

The experience of the pandemic may conversely have opened people's eyes to the benefits of adopting eHealth, and act as an incentive for adoption. Over the last three years, there has been a universal shift in our attitudes towards use of technology.^33^ We have become more receptive to incorporating new uses into our daily lives. In care homes, use of eHealth played a vital role in facilitating communication with external services, and maintaining person-centered care delivery.^34^ The intention of the Framework is to empower care home staff without healthcare training to assess and administer care more confidently, and communicate effectively with healthcare professionals—features which would have been hugely beneficial during ‘lockdown’ times. Implementing the EMBED-Care Framework in a post-COVID landscape to a more eHealth literate population,^35^ with an understanding from experience of the advantages of working in this way, may in practice be less of a challenge than anticipated.

Previous implementation research has emphasised the need to engage care home leadership, if an intervention is to be successfully adopted.^36^ Whilst the commitment of care home managers was acknowledged as key, whether this has real-world influence on other staff members’ interest was questioned by workshop participants. Rather, the merits of a ‘bottom-up’ approach involving the individuals who provide direct clinical care to residents was advocated. This echoed a key finding from the systematic review which informed this study, regarding the need to involve all staff prospectively and throughout the implementation process. We termed this ‘engaging end-users’^12^ and have honoured this here through using co-design, with health and social care staff across disciplines and grades, to ensure a sense of shared ownership and strengthen the credibility of the findings. We will continue this inclusive approach throughout the planned feasibility study.

A common finding in existing literature is the importance of considering the organisational context in which an intervention is to be implemented, and how the determinants and strategies required might vary between different settings, for example, residential versus community care.^37^ This becomes increasingly complex in the case of care homes: as demonstrated here, much variation exists even within the care home setting. Disparities regarding resources, technology in use, routine work process and communication channels with external practitioners are serious practical challenges when planning implementation both within and across multiple care homes. Divergence between preferences regarding aspects of intervention delivery was highlighted in the workshops, for example, how to involve family in care in a way that is meaningful and reassuring whilst minimising burden.

The practical implication of this is the need to tailor implementation of the Framework to the individual needs of the residents, staff, families and care homes and ensure elements of it are adaptable to each local context. Maximising compatibility and ease of use between the Framework and the individual care home systems, and not disrupting or duplicating processes, is key if it is going to be adopted. Distinguishing between which elements of the Framework are modifiable to the local context, and what should be delivered as manualised to maintain use, will be pursued in the upcoming feasibility study of the EMBED-Care Framework.^38^

Although crucial to consider the individual care home setting, the external influences—such as policy requirements—on the success of implementation should not be overlooked. Changes in national guidance can facilitate implementation of a new way of working if it aligns with newly introduced recommendations. In the case of the EMBED-Care Framework, requirements around adopting a digital infrastructure and conducting a holistic assessment within one week of admission as laid out in the recently introduced NHS policy ‘Enhanced Health in Care Homes’ (EHCH)^39^ may encourage uptake, and this synchronicity should be promoted as part of the implementation plan.

Ensuring alignment with policy can be a challenge; guidance can change rapidly, as observed during the pandemic.^40^ However, it is a vital consideration which may influence uptake of the EMBED-Care Framework, and we must engage with policy and policy makers prospectively to enhance impact. Strengthening this relationship is also potentially beneficial for policy makers; working with implementation experts could act to enhance receptivity of new policy, to bridge the gap between policy-intent and implementation.^41^

### Implications for policy and practice

Using our findings, we have developed a theoretically informed implementation plan comprising multiple strategies to target the overarching principles of successful implementation of eHealth for use in care homes for people with dementia (see Table 3). Strategies which corresponded with implementation determinants we identified at the three phases were selected from a published compilation of 73 implementation strategies developed by a panel of implementation experts, as part of the Expert Recommendations for Implementing Change (ERIC) project.^42^

**Table 3.** Strategies for implementing eHealth for use with people with dementia in care homes.

Phase	Strategies	Explanation in context of eHealth
Incentivising individuals to adopt the intervention	Develop and distribute educational materials	In line with findings stressing the importance of promoting the value of eHealth, materials (e.g. newsletter bulletins, flyers, social media posts and PowerPoint presentations) should be developed and circulated to potential sites to highlight the likely benefits that adopting the new way of working might have, to maximise engagement (e.g. to facilitate communication, improve care procedures, streamline workload)*.* Comprehensive training materials should be developed and distributed to all users. These should be a mix of materials, including, for example, a glossary, FAQ'S, a training manual and pre-recorded training videos.
	Identify and inform local opinion leaders and influencers	This is a key part of the site recruitment strategy and relates to informing individuals who are ‘educationally influential’ about the intervention, encouraging them to influence others to adopt it. In England, this includes identifying representatives from the National Institute of Health and Care Research (NIHR) Clinical Research Network's (CRN), and the NIHR ENRICH (Enabling Research in Care Homes) network of ‘research ready' care homes, to help identify and recruit potential sites and care home forums. Contact with other multi-disciplinary team members who might be involved in using the Framework, including district nurses and local GP's.
	Conduct educational outreach visits to encourage teamwide change	Implementation requires a whole-team approach and a sense of shared responsibility. Once sites are identified, a meeting should be held initially with care home management to explain the purpose of the eHealth intervention, what participating in the study would entail for all staff members and what support can be provided. Time and consideration must also go into careful explanation of what the requirements and benefits of enacting the intervention would be to all remaining staff, for example, professional development and improved work processes, to encourage collective commitment to its use.
	Involve residents and family members in the implementation effort	Involving users and stakeholders prospectively throughout development of the intervention and implementation plan is key to ensure a sense of shared ownership, increase the credibility of the intervention and promote its uptake. Prioritising the needs and wants of the resident and involving their family members is a hallmark of good quality palliative care. Where possible, the person with dementia should be encouraged to be involved in all care decisions. To accommodate varying preferences regarding the extent to which family would like to be involved, levels of communication concerning the resident should be tailored to the preference of the family and clarified prior to implementation.
	Access new funding	Cost is a major barrier to implementing eHealth in care homes. This can be alleviated by providing reimbursement for care homes for staff time. Reimbursement should be offered for meetings with management to initiate site set-up, staff training and for any ongoing additional duties which will be required of them.
	Ensure alignment with regional and national priorities, and regulations, for example, data sharing	Implementation of the eHealth intervention will be facilitated if its aim is supported by external recommendations and acts to address regional and national priorities. Implementers should work closely with policy makers to ensure alignment of the intervention with current policy and guidance to enhance impact of the intervention, and synchronicity between the two should be promoted as part of the implementation plan. eHealth also needs to comply with national requirements to ensure it complies with, for example, GDPR and information sharing.
Ensuring individuals can enact the intervention	Promote adaptability and tailor strategies	Tailoring the intervention to the both the setting and user will be essential for its uptake and to optimise compatibility. To maximise adoption, sites should be encouraged to utilise the intervention in a way that is most compatible with their current practice and the individual residents, for example, regularity and timing of assessment, how information is communicated with families and external health care professionals. Flexibility of the intervention should be promoted, whilst being mindful of maintaining its fidelity.
	Assess for readiness and identify barriers and facilitators	A baseline assessment of each individual care home should be conducted so that the intervention can be tailored to the site's needs. Care home management should be met with in advance to ensure that they have sufficient resource to support the intervention, for example, bandwidth and tablets (which should be provided if not available); and to learn about their current routine practice, for example, arrangement with external health care professionals, communication routes, MDT meeting schedules and current training processes. Research readiness should be assessed prior to implementation using an appropriate measure, for example, the Holt Readiness for Organisational Change Tool,^46^ which measures features of the organisational context that might impact a care homes’ capacity to implement new ways of working, for example, leadership styles.
	Remind practitioners	Uptake of the intervention can be facilitated by introducing a reminder system designed to prompt users to initiate the intervention. Diarized reminders should be tailored to each resident dependent on need. Electronic alerts should notify either when an assessment should be initiated, or if a symptom or concern is identified in the assessment.
	Identify and prepare champions	Two or three staff ‘champions’ of varying grades and experience should be identified in each care home by the care home manager to drive implementation of the intervention, deliver training, monitor implementation and support other users. Certificates should be provided to recognise, value and incentivize participation in a champion role.
	Conduct educational outreach visits	Multiple methods should be used to ensure that sufficient training is provided for all users. Emphasis should be placed on the ability of training to benefit staff career progression, and empower them to escalate concerns. Where possible, training should be delivered to both health and social care workers together. Prior to implementation training and support will include: A pack of training resources including a user-manual, a glossary and contact details to access technical support (phone number and email). At least one face-to-face interactive training session should be offered to all staff. A series of ‘bitesize’ instructional videos built into the app should also be provided, so they can be easily and regularly accessed. Further training should be delivered to two or three ‘champions’; individuals who have been identified to lead the intervention in the individual care setting, and drive it forward in absence of the research team. Certificates should be provided to staff on completion of training for personal development records.
Ensuring individuals sustain use of the intervention	Audit and provide feedback on the impact of using the Framework	Ongoing evaluation of progress is vital in order to appraise the impact that an intervention is having, and to ensure it is effectively embedded. Data should be collected and summarised over the implementation period and fed-back to staff, families and residents. Data should include usage of the app and any impact it is having on symptom management, quality of life measures and carer burden. This can be relayed in staff meetings, or via newsletters or email. Graphs depicting changes in measures, for example, resident symptoms over time should also be made available and circulated to stakeholders.
	Purposely and regularly re-examine and evaluate outcomes of the implementation and opportunities to strengthen use in clinical care	Uptake of the intervention should be closely monitored over the implementation period, and strategies adjusted throughout the process to promote continuously its sustained use and improve the quality of care. Short weekly meetings with champions should take place to understand ongoing experience of using the intervention, to monitor implementation and identify and rectify problems. The implementation strategies should be reviewed regularly and refined, for example, if there a need for more education/training, if the adaptable component is working, if the reminders are occurring at sufficient frequency etc. Care records can be cross-referenced with app-usage data to infer if the intervention is leading to a change in care, for example, in medication, referrals to external services. Focus groups and interviews should be conducted at the end of the implementation period to explore if and why a change in care was or was not observed.

As the ERIC strategies^42^ are largely focused on implementing an innovation which has already been developed, there was no existing strategy to correspond with our findings around ensuring the intervention in development is aligned with external best practice recommendations. We have therefore created an additional strategy, titled ‘Ensure alignment with regional and national priorities’, to capture findings around requirements to ensure compatibility between the intervention and national guidance or policy, and to facilitate uptake.

Given our consistent findings around the importance of a participatory approach to implementation, requiring engagement and commitment to change from staff of all levels of seniority we have revised the ERIC strategy named ‘Mandate change’ to ‘Encourage teamwide change’, increasing applicability to this context.

This paper presents an in-depth co-design development of an implementation plan for a novel eHealth intervention to support holistic assessment and decision making. Although created with the EMBED-Care Framework in mind, the phases outlined here provide useful findings beyond this specific intervention and its lessons can be applied more generally to implementation of eHealth for use with people in dementia in care homes. The systematic review which formed the foundation of this work explored implementation requirements of eHealth for people with dementia in long-term care, through a wider lens. Further, as the intervention and implementation plan were developed simultaneously, the specifics of the intervention had not yet been finalised at the time of the workshops. As such, this context-specific multi-strategy plan can be used as a practical guide for individuals attempting to implement other eHealth interventions in this setting with this population.

### Strengths and limitations

The strategies developed in this paper have a strong theoretical basis, and target empirically demonstrated mechanisms of change.^24^ Our novel use of a co-design methodology in their development enabled us to ensure that they are relevant, viable and responsive to user needs. The revision of the ERIC strategies and NPT to accommodate the context in which the Framework is being implemented furthers the scope of the theory, increases its applicability to this particular setting and population, and its relevance in the post-COVID landscape.

Due to COVID-19, the workshops were conducted remotely over video-conferencing software. There are clear disadvantages to this, including loss of elements of personal interaction and the onset of ‘zoom fatigue’.^43^ However, virtual workshops allowed for recruitment from a wider and thus more representative base of participants from across the UK.

Workshop participants included a mix of healthcare, social care practitioners and family carers and there were often occasions where opinions differed. Balancing contradictory priorities and overcoming hierarchical power structures was a challenging element of facilitating workshops, however, eased by the use of video-conferencing software. Research has highlighted that participants who feel apprehensive to speak may be more comfortable participating when attending meetings remotely,^44^ providing a more varied and richer discussion.

A limitation of this study is the lack of formal inclusion of people with dementia as study participants. Although intended, due to lockdown restrictions and the difficulties of conducting workshops with this population virtually, we were unable to include them as planned. To rectify this, when able we ran a subsequent series of public engagement workshops for people with, and personally affected by, dementia. In these, we presented the Framework and discussed potential implementation strategies, to ensure the viewpoints of people with dementia were adequately represented in development of the plan.

## Conclusion

Implementing eHealth into such a complex system of care is a multifaceted and challenging process, which requires collaboration with stakeholders in order to develop a valuable and credible implementation plan, with real-world relevance. Together, we identified three overarching phases of implementation with distinct requirements to be met, for implementation to be successful. Underpinned by the NPT, we have developed a theoretically driven multi-strategy plan to target mechanisms known to shape implementation process and outcomes. This can be employed when implementing eHealth to improve care processes for people with dementia in care homes.

## Supplemental Material

sj-docx-1-dhj-10.1177_20552076231211118 - Supplemental material for Co-design of a theory-based implementation plan for a holistic eHealth assessment and decision support framework for people with dementia in care homes Click here for additional data file. Supplemental material, sj-docx-1-dhj-10.1177_20552076231211118 for Co-design of a theory-based implementation plan for a holistic eHealth assessment and decision support framework for people with dementia in care homes by Juliet Gillam, Catherine Evans, Jesutofunmi Aworinde, Clare Ellis-Smith, Jamie Ross and Nathan Davies in DIGITAL HEALTH

sj-docx-2-dhj-10.1177_20552076231211118 - Supplemental material for Co-design of a theory-based implementation plan for a holistic eHealth assessment and decision support framework for people with dementia in care homes Click here for additional data file. Supplemental material, sj-docx-2-dhj-10.1177_20552076231211118 for Co-design of a theory-based implementation plan for a holistic eHealth assessment and decision support framework for people with dementia in care homes by Juliet Gillam, Catherine Evans, Jesutofunmi Aworinde, Clare Ellis-Smith, Jamie Ross and Nathan Davies in DIGITAL HEALTH

sj-docx-3-dhj-10.1177_20552076231211118 - Supplemental material for Co-design of a theory-based implementation plan for a holistic eHealth assessment and decision support framework for people with dementia in care homes Click here for additional data file. Supplemental material, sj-docx-3-dhj-10.1177_20552076231211118 for Co-design of a theory-based implementation plan for a holistic eHealth assessment and decision support framework for people with dementia in care homes by Juliet Gillam, Catherine Evans, Jesutofunmi Aworinde, Clare Ellis-Smith, Jamie Ross and Nathan Davies in DIGITAL HEALTH
