# Supplementary material for: Co-design of a theory-based implementation plan for a holistic eHealth assessment and decision support framework for people with dementia in care homes
Source: Digit Health. 2023 Nov 28;9:20552076231211118. doi: 10.1177/20552076231211118 (PMC10685752; doi:10.1177/20552076231211118)
Supplement: sj-docx-2-dhj-10.1177_20552076231211118 - Supplemental material for Co-design of a theory-based implementation plan for a holistic eHealth assessment and decision support framework for people with dementia in care homes [file sj-docx-2-dhj-10.1177_20552076231211118.docx]

**Workshop outline**

**Workshop one**

- Implementation was introduced: key definitions, and why it’s important when developing alongside a complex intervention
- The intervention prototype was introduced to the group
- The group was presented with characteristics of eHealth which had been identified in the previously conducted systematic review to facilitate its implementation: 1) simple and user friendly, 2) portability, 3) customisability, 4) including alerts and reminders, 5) compatibility with other systems, 6) the ability to provide useful feedback on its impact to users
- *Question*: How might we go about incorporating these features into our eHealth intervention? E.g. what would user-friendly look like to you?
- *Question*: What other key features could the eHealth device have that might increase its chances of uptake?

**Workshop two**

- Re-capped on the previous workshop: we reiterated what implementation is, and why it’s important in health and social care research. The updated intervention prototype was presented.
- *Question*: What factors might influence whether you are likely to use the EMBED-Care intervention in your setting?
- Once identified, the group sorted these factors into what might facilitate, or be a barrier to, implementation.
- The group was presented findings from the systematic review around what factors have been previously identified to be a barrier to eHealth implementation: 1) lack of staff time, 2) concerns around patient data, 3) lack of resource/training, 4) disruption to work routine, 5) complexity of eHealth.
- We discussed how we could apply these to the EMBED-Care intervention, and the importance of compatibility with current practice – in order to be adopted, eHealth must be compatible with existing routine practice and align with the values of the care setting.
- *Questions:* 1) What is the usual process of assessment? 2) do you currently use eHealth? 3) how might eHealth disrupt normal processes? 4) what barriers might you encounter if you were implementing the EMBED-Care intervention in your setting? What barriers did you identify? How could these barriers be overcome?
- We then focused on how we make sense of the new intervention - in order to be used, people must make sense of the intervention and understand its unique value and advantage over how care is currently provided.
- *Questions*: 1) How would using the intervention change the way you approach managing the symptoms of someone with dementia? a) assessment b) decision making 2) how might it enhance current practice? 3) what key things would you like to know and understand?
- The group was presented with suggestions from systematic review around what the benefits of using this type of intervention might be: 1) to give carers permission to administer care and to escalate to GP, 2) to bring together complex information in one place, 3) reduce unplanned admissions to hospital from the care home, 4) streamline and co-ordinate care.
- How these might apply to the EMBED-Care intervention, along with other suggestions from the group was then discussed.
- *Question:* What strategies can we use to ensure that end-users are aware of the benefits, and how can we promote it?
- The group was presented with suggestions from systematic review: 1) to contact care home managers and other influential individuals 2) to conduct educational meetings e.g. training staff, residents and family members 3) to develop and distribute educational materials e.g. manuals & glossary, social media, technical assistance.
- How we could apply these to the EMBED-Care Framework was then discussed.

**Workshop three**

- Recapped the previous workshop discussion.
- Focused on the importance of engaging end-users, staff members, family carers and people with dementia with the intervention being crucial for uptake; from buy in to committing to sustain the new practice.
- *Question*: How can we engage stakeholders?
- The group was presented with suggestions from systematic review: 1) approaching care homes, 2) engaging care home management, 3) identifying champions, 4) tailoring implementation protocols 5) providing sufficient training.
- *Question*: How can we apply these to the EMBED-Care Framework?
- Discussed training requirements: Care home staff, family carers and the people with dementia will receive training before using the intervention in practice.
- The group was presented with suggestions from systematic review around what good training might look like: 1) Joint working with an expert, 2) training on the job vs. before implementation, 3) train-the-training approach, 4) training manuals, 5) eLearning.
- *Questions*: Do you think these would work with EMBED-Care? What has worked in the past from your experience?
- Focused on evaluating the intervention: being able to observe and appraise the outcomes that implementing a new way of working is having is key for continued engagement.
- *Questions*: What can we put in place to ensure that individuals sustain use of the intervention?
- The group was presented with suggestions from systematic review: 1) usage data collected through eHealth tool, 2) demonstrating the intervention is not just another “tick box”, 3) showing an improvement in carer burden measures or quality of life, 4) providing a graph with changes in scores over time.
- We discussed how these might be applied to EMBED-Care, and any additional suggestions on how we could appraise the intervention.
