# Supplementary material for: Co-design of a theory-based implementation plan for a holistic eHealth assessment and decision support framework for people with dementia in care homes
Source: Digit Health. 2023 Nov 28;9:20552076231211118. doi: 10.1177/20552076231211118 (PMC10685752; doi:10.1177/20552076231211118)
Supplement: sj-docx-3-dhj-10.1177_20552076231211118 - Supplemental material for Co-design of a theory-based implementation plan for a holistic eHealth assessment and decision support framework for people with dementia in care homes [file sj-docx-3-dhj-10.1177_20552076231211118.docx]

**Table 1**: Quotations illustrating theme one: Incentivising adoption.

| **Quotations illustrating theme one: Incentivizing adoption of the Framework** | |
| --- | --- |
| **No.** | **Quote** |
| **1** | *It allows the person living with dementia to be at the centre. It involves care home staff who know the individual, often very very well, and it involves their residents’, important others as well… [GP, workshop three]* |
| **2** | *I think it might help them [care staff] to identify when things need to be done. If you see somebody every day you don’t always notice subtle changes, do you?*  *[Hospice education lead, workshop two]* |
| **3** | *It's not always recognized how important - how … incredibly important - the psychological welfare of the patients is; well, I don't think it's always recognized 'cause the focus is so much on the very end of life care that is provided...*  *[Hospice education lead, workshop two]* |
| **4** | *But I think if you could somehow integrate the system so if your person with dementia is in a care home environment the family almost have access to that information as well… if they have to sort of update a record on an app, which is accessible by the family as well as clinicians … that would be a huge sense of comfort.  [Family carer, workshop one]* |
| **5** | *I think it's going to be very much ‘horses for courses’ and what people can cope with 'cause of the emotional upset for you in that triangle, the person, the family carer, the practitioners.  (Nurse specialist, workshop one)* |
| **6** | *…a lot of the residents are kind of feeling that that they'd [been] deserted, let down, abandoned and all those kinds of things. [Care home manager, workshop two])* |
| **7** | *I think everyone is experiencing difficulties accessing primary care at the moment… It's obviously very, very hard for everybody, and there's a backlog of care in the sense that hospitals were shut essentially to non-COVID patients for a significant period of time. [Consultant psychiatrist, workshop two]* |
| **8** | *So I think the manager, convince the manager… Because they are the key point, because you actually might have staff interested but sometimes, my question is, if the message passes from the managers to the staff.  [Palliative Care Clinical nurse specialist, workshop three]* |
| **9** | *You know, so I think this is the sort of thing that works really well as a sort of bottom up approach. So if you've got people on the floor who are providing that direct clinical care to the people living with dementia, then I think there's a lot more value in investing the time and the energy in supporting them really. [Advanced clinical practitioner, workshop two]* |
| **10** | *And articulating their concerns across to the ambulance crews, 111, GPs or their local sort of acute response teams, it just gives a little bit more understanding around how to emphasize and how to escalate [care interventions]… So I think things that empower staff in order to update their point about somebody (would really help).  [Advanced clinical practitioner, workshop three]* |
| **11** | *And I think your point about benefiting the staff, particularly the care home staff and the GP's, is a really good one because my observation is that it's very easy to suggest a small change to care home staff .. but that one small change is in addition to other lots of small changes… change fatigue? .. adding in another task that's going to be perceived as additional work.. [Consultant psychiatrist, workshop two])* |
| **12** | *Think about how could you sell it to a primary care network that's reaching new standards, “say OK, well, we don't think you are because you're not doing XYZ for people with dementia. Going up with us, engage your care homes and there you go, you can take that”…I think it's about making it easy for people, isn't it? Appeal to their ambitions, 'cause people genuinely want to provide better care.. [GP, workshop three]* |
| **13** | *The CCG's [Clinical Commissioning Groups] are changing out there from April next year [re-configured as Integrated Care Systems]. And it's kind of a bit difficult to get straight answers, but you know they don't know what's going to be happening and how things are going to work. So to try and instigate something new at the minute coming from that kind of level from the GP or the community teams I think will be quite difficult, yeah? [Hospice education lead, workshop two]* |
| **14** | *I do think that there's still are people out there who find technology frightening. And you know, to be inputting into a device. You know, it might.. it might trigger anxieties (Nurse specialist, workshop one)* |
| **15** | *But I always think it becomes very depersonalized, because we are doing the obs [observations] and then we're putting the things in the screen, and we're not really even focusing on the patient a lot of the time and so that's often a barrier. (Registered nurse, workshop one)* |
| **16** | *My only caution would be around permissions and, data protection. Yeah, you know: if a person is able to consent to their data being shown. We would need any data sharing needs to be very, very carefully considered. (GP, workshop one)* |

**Table 2:** Quotes illustrating theme two: Enabling operation of the Framework.

| **Quotations illustrating theme two: Enabling operation of the Framework** | |
| --- | --- |
| **No.** | **Quote** |
| **17** | *It's around avoiding duplication, isn't it? If you're asking the care or the nursing staff to do these assessments, it's got to fit in with whatever system they use;, and so if they're using an electronic system of assessment, it needs to fit in well with that and not duplicate what they're already doing.  [Hospice education lead, workshop two]* |
| **18** | *.. obviously you know not all homes are equipped with Wi-Fi, nor do they have signal. (Nurse specialist, workshop one)* |
| **19** | *It's very sketchy around the country. I think you've got some areas where they've got excellent links with community professionals and then other areas where things are changing so much ... [Hospice education lead, workshop two]* |
| **20** | *I think it's probably case by case. Isn't it? Because needs will be so different. So I think I think the need for it to be very flexible in how it can be set up*  *(Nurse specialist, workshop one)* |
| **21** | *..but I think if you think about a care home, one, care home or two care homes, they're nearly all in the same primary care network… Usually got the same district nurses… the same team with Admiral nurses, the same small group of GP’s. Fairly consistently, you know there's a really consistent group of people.. (GP, Workshop three)* |
| **22** | *Yeah, I was thinking : if there's some alert .. like if someone’s diet has changed dramatically, or if someone was mobile and now they’re chair bound or bedbound, things like that.. to remind us the care plan needs to be updated to (include) those major changes  [Family carer, workshop one]* |
| **23** | *It has to be very simple; it has to be something that the person living with dementia will be able to use, and their carers would be able to use, and they don't have to be worried about how to use it. (So it has to be very simple, very user friendly.) [Family carer, workshop one]* |
| **24** | *…also maybe include a sort of almost like a dictionary or something, to give you some meanings for some of the words that you may have come across from practitioners dealing with you, or your family relative. (Family carer, workshop one)* |
| **25** | *So, trying to build some champions within the team … Actually, you know if you’re going to bring the tool in, it’s about the constant monitoring [of symptoms and concerns, and outcomes of care] isn’t it? … Even if everybody says it's a great idea, you’ve actually got to have that constant motivation and enthusiasm.)*  *[Hospice clinical lead, workshop two]* |
| **26** | *I think also if someone like the champion you've got someone who to go to, to ask if you're not sure. There's been quite a significant turnover of staffing. It is really hard work, and you know, again, it can be tricky getting used to a new electronic system. And I need there to be someone there to ask.  [GP, workshop two]* |
| **27** | *I think the videos [to support training] are a good idea. It needs to be a blended approach, doesn't it? Really, so that you're getting the information across in in different formats  [Hospice education lead, workshop three]* |
| **28** | *I think I mentioned it in the chat: doing things in bite sized manageable chunks because again, with staffing pressures ..I think if you do it in smaller bite sized manageable chunks it makes it more accessible for people.  [Advanced clinical practitioner, workshop three]* |
| **29** | *Care home staff I meet, really take pride in their work. And they're really grateful for provision of training because if it's part of career progression, and learning how to deliver their job better, I think lots of people would take it up. [GP, workshop two]* |
| **30** | *They begin to understand each other's challenges … they have a new respect for other professionals, other agencies.  [Hospice education lead, workshop two]* |
| **31** | *Maybe to use a fancy term “parity of esteem” which mental health trusts bang on about but… I think workers within social care are perhaps left behind in comparison to those working [in the NHS], and if you deliver the training to them alongside each other. [GP, workshop two]* |

**Table 3:** Quotes illustrating theme three: Sustaining use of the Framework.

| **No.** | **Quote** |
| --- | --- |
| **32** | *...People have got to feel the benefit. “What's in it for me?” For this thing to happen, you know whether I'm the patient or the family carer, 'cause they're the people you strongly need the buy in from. “What's the saving?” (Nurse specialist, workshop one)* |
| **33** | *And they can see the advantages of using it and what they're going to gain from it? Sometimes I feel the staff feel like they're doing it but nothing really comes from it.. We kind of still need to remind them of the changes.. People probably will start thinking “What's the point?” [Clinical nurse specialist, workshop three]* |
| **34** | *Maybe if some data could be collected where they can actually see the scores going down after their interventions, that can show the benefit of it. [Clinical nurse specialist, workshop three]* |
| **35** | *I think that's [a graph plotting staff use] a very nice idea, and you can maybe even give them a monthly report, “look how well you did”. A Monthly report to the managers.  [GP, workshop three])* |
| **36** | *Facebook's, you know, free, but it's really nice to see what they're doing in the care homes, so I suppose that would be a way of getting it out to them. [Hospice education lead, workshop three]* |
| **37** | *So I've realized that actually small, small steps are the easiest way to do it… I think what we also do routinely in healthcare is “let's just do an additional thing”, rather than stopping and thinking.  [Care home activities co-ordinator, workshop two]* |
| **38** | *I would have thought that [ feedback on staff use of app] would be very useful because you're also talking about building up a new set of habits… but it's then in months 23 and 24 are they still being used ? (It’ll be necessary to) underline that this should be used, and used regularly, so that I would have thought (that’d be) a very important way of monitoring and changing habits and changing procedures with it. [Hospice education lead, workshop two]* |
